# Towards Intelligent Wound Care: Hydrogel-Based Wearable Monitoring and Therapeutic Platforms

**DOI:** 10.3390/polym17131881

**Published:** 2025-07-06

**Authors:** Yan Niu, Ziyao Zhao, Lihong Yang, Dan Lv, Rui Sun, Ting Zhang, Yuhan Li, Qianqian Bao, Mingqing Zhang, Lanzhong Wang, Wei Yan, Fei Han, Biwei Yan

**Affiliations:** 1 Key Laboratory of Agricultural Microbiology in Gansu Province, College of Bioengineering and Biotechnology, Tianshui Normal University, Tianshui 741000, China; 2 The Key Laboratory of Biomedical Information Engineering of Ministry of Education, School of Life Science and Technology, Xi’an Jiaotong University, Xi’an 710049, China; 3 Bioinspired Engineering and Biomechanics Center (BEBC), Xi’an Jiaotong University, Xi’an 710049, China

**Keywords:** hydrogel-based wearable technology, wound monitoring, multifunctional wound dressing, closed-loop feedback, chronic wound management

## Abstract

Chronic wounds present clinical challenges due to persistent inflammation, infection, and dysregulated tissue repair, often exacerbated by the passive nature of conventional wound dressings. Recent advancements in hydrogel-based wearable technologies have transformed these biomaterials into multifunctional platforms capable of integrating real-time monitoring and targeted therapy, ushering in a new era of intelligent wound care. In this review, we show innovative diagnostic and therapeutic strategies, including wound-monitoring devices and multifunctional healing-promoted platforms, highlighting integrated closed-loop systems that dynamically adapt treatments to wound microenvironments, thus merging diagnostics and therapeutics. Challenges in fabrication engineering and clinical application are discussed, alongside emerging trends like AI-driven analytics and 3D-bioprinted technology. By bridging fragmented research, this work underscores the potential of hydrogels to enable intelligent wound management.

## 1. Introduction

Wound healing is a prolonged process due to many issues like ongoing inflammation and poor tissue regeneration and remodeling, especially in chronic wounds [1,2]. For instance, diabetic foot ulcers (DFUs) are a common problem that affects about 18.6 million individuals with diabetes worldwide in 2023. These wounds often become chronic and cannot heal easily, leading to disability in about 20% of cases and even death in roughly 30% within five years [3]. Traditional dressings mostly act as passive covers by protecting the wound and soaking up excess fluid, but they do not actively help it heal [4,5]. To improve this, an active wound-care system is needed that can both monitor the wound environment in real time and deliver optimal treatments [6,7]. For patients, such integrated wound-care systems can meet their specific demands. Doctors can optimize treatments more accurately by using ongoing feedback, which could provide faster healing, fewer complications, and lower costs.

Hydrogels have developed dramatically since their early development, transitioning from water-absorbent polymers to multifunctional biomaterials for various biomedical applications [8,9]. With recent developments in polymer science, the application of hydrogels has expanded largely. For example, when used as wound dressings, they not only provide a physical barrier and a moist microenvironment for wound, but they also provide an excellent performance, including an inherent biocompatibility, tunable mechanical properties, and the ability for functionalization with bioactive molecules [10,11]. These characteristics have made hydrogels ideal candidates for an integrated wound-care system.

To overcome the limitations of traditional wound care, there is a promising strategy to integrate the diagnosis with the treatment within a hydrogel-based wound dressing [12,13]. However, most of the current research has mainly focused on either the diagnostic or therapeutic functions rather than an integrated wound-care system integrating a diagnosis [14,15] and therapy [16,17]. This disconnection hinders the development of wound-care solutions that can dynamically respond to the wound microenvironment. Thus, there exists a critical need for a comprehensive review to highlight the gaps between the current treatments and the real needs for wound care.

**Figure 1:**
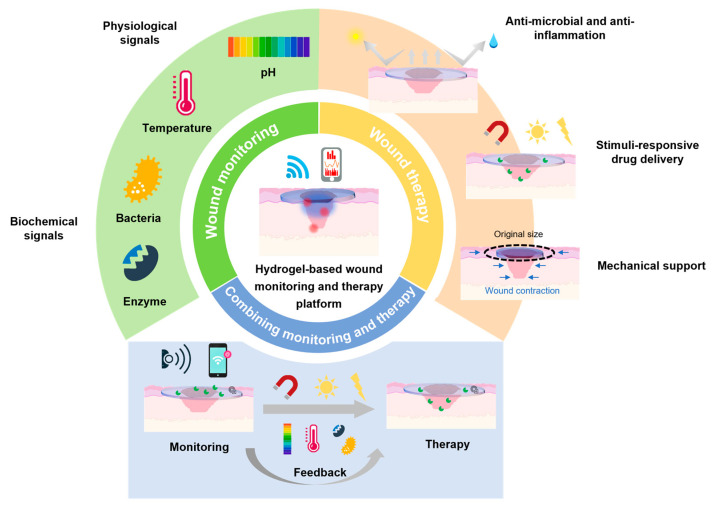
Schematic illustration of hydrogel-based wearable monitoring and therapeutic platform.

The objective of this review was to provide a comprehensive analysis of integrated wound-care systems based on polymer hydrogels, with a focus on merging diagnostic and therapeutic functionalities into a single platform (Figure 1). In this context, the review is structured into three main sections: (1) an introduction to the fundamentals of wound healing and the role of hydrogels, including their polymer chemistry, manufacturing strategies, and critical performance, (2) a demonstration of innovative diagnostic and therapeutic strategies, including wound-monitoring devices and multifunctional healing-promoted platforms, and (3) a discussion of how these diagnostic and therapeutic components can be combined to create an integrated wound-care system. This review aimed to clarify the current challenges in wound care, discuss the potential solutions, and identify future directions in the development of intelligent hydrogel-based wound-care strategies.

## 2. Fundamentals of Wound Healing and the Role of Hydrogels

### 2.1. Wound-Healing Physiology

Wound healing initiates with hemostasis, with a reduced blood flow through vasoconstriction and thrombosis through platelet aggregation. Then, these platelets simultaneously release growth factors and immune cells [18]. During inflammation, neutrophils firstly eliminate pathogens by releasing reactive oxygen species (ROS) [19,20]. Then, macrophages replace neutrophils, transforming from pro-inflammatory to anti-inflammatory states. Over days to weeks, a proliferative phase is initiated, including re-epithelialization, collagen synthesis, and angiogenesis [21,22]. Finally, fragile type III collagen is replaced by stronger type I collagen in the remodeling phase, providing mechanical robustness for repaired tissues [23,24].

Wound biomarkers play a pivotal role in evaluating the wound status by offering information about the wound microenvironment. Physiological parameters such as the temperature, oxygen levels, and humidity levels can provide direct feedback about the wound’s local conditions. For instance, an elevated temperature reflects inflammation [25], while hypoxia indicates impaired perfusion [26]. The biochemical parameters mainly include the pH, metabolic biomarkers (e.g., glucose, uric acid), cytokine (e.g., IL-6, TNF-α, IL-10), enzymes, and microbes. For example, an acidic pH may cause an infection [27], and hyperglycemia suggests diabetic dysregulation in chronic wounds [28].

### 2.2. Polymer Chemistry of Hydrogels

Hydrogels can be generally classified by their source, and can contain natural, synthetic, or hybrid polymers with distinct advantages and limitations [29]. Natural polymers can mimic biological environments because of their inherent biocompatibility, biodegradability, and bioactivity, including hyaluronic acid [30], chitosan [31], alginate [32], gelatin [33], and collagen [34]. However, their poor mechanical stability hinders their broad use, which can be solved by introducing synthetic polymers, such as polyacrylamide (PAAm) [35], polyethylene glycol (PEG) [36], or polyvinyl alcohol (PVA) [37], with tunable mechanical properties and chemical stability. To integrate their advantages, hybrid polymers like gelatin–methacryloyl (GelMA) [38] and silk fibroin–PEG composites [39] have been developed, with the bioactivity of natural polymers and the stability of synthetic polymers. They play an important role in biomedical applications of hydrogels.

In addition to the polymer source, hydrogels can be categorized by their crosslinking mechanisms, which largely determine their structural and functional properties [40]. Physical crosslinking uses non-covalent interactions such as hydrogen bonds [41], ionic linkages [42], or crystallite formation [43], enabling rapid gelation in clinical applications. In contrast, chemical crosslinking is based on covalent bonds formed via photo initiators [44] or enzymatic reactions [45], yielding mechanically stable networks suitable for long-term wearable devices. In addition, dynamic or reversible crosslinking employs stimuli-responsive bonds (e.g., Schiff base [46], boronate esters [47], or host–guest interactions [48]) to develop self-healing hydrogels, which autonomously repair mechanical damage, enhancing their durability. Each strategy balances trade-offs between the gelation speed, stability, and adaptability, so a customized hydrogel design is necessary for each specific biomedical application.

Based on optimal crosslinking strategies, hydrogels are endowed with excellent structural properties. Advanced functionalization is further needed to tailor these materials for smart wound management, mainly including conductive additives [49], bioactive moieties [50], and stimuli-responsive components [51]. Hydrogels with conductive additives (e.g., MXene [52], polypyrrole [53], or PEDOT:PSS [54]) enable the real-time sensing of biophysical signals for wound progression monitoring. In addition, bioactive moieties like grafted VEGF [55], antimicrobial peptides [56], or glucose oxidase enzymes [57] endow hydrogels with some biochemical functions, such as angiogenesis, infection control, or metabolic regulation. In addition, hydrogels with stimuli-responsive components can provide smart wound management according to the wound microenvironment through dynamic interactions (e.g., pH-sensitive carboxyl groups [58,59], thermo-responsive poly(N-isopropyl acrylamide) (PNIPAAm) [60], or light-activated spiropyran units [61]). For instance, PNIPAAm hydrogels can modulate drug release through temperature-dependent swelling [62], while spiropyran can enable on-demand antimicrobial activation by the photochromism effect [63].

### 2.3. Manufacturing Strategies and Critical Performance of Hydrogels

The fabrication of hydrogels has developed from traditional molding techniques to advanced microfabrication and additive manufacturing approaches, which aim to enhance the precision, functionality, and integration of multiple components (e.g., biosensors and drug reservoirs) within the hydrogel matrix [64,65]. The manufacturing method directly affects the architecture, responsiveness, and applicability, mainly including self-assembly [66], screen-printing [67], 3D-printing [68], electrospinning [69], and micromachining techniques [70]. Self-assembly technology relies on non-covalent interactions, in which basic structural units (e.g., molecules, nanomaterials, and micrometer or larger-scale materials) spontaneously form an ordered structure [71]. It is particularly useful for injectable hydrogels and stimuli-responsive drug release due to its simplicity and minimal equipment requirements [72,73]. Screen printing is a scalable, low-cost fabrication method for patterning conductive inks or hydrogel precursors onto flexible substrates, which enables the fabrication of functional layers such as enzyme-based biosensors [74,75]. Three-dimensional printing offers precise control over the hydrogel architecture at the microscale level. It is ideal for patient-specific wound dressings and drug-loaded scaffolds [76,77]. Electrospinning produces nanofibrous hydrogel membranes with a high surface area and ECM-mimetic properties, supporting cell adhesion and controlled drug release [78,79]. Micromachining techniques (e.g., soft lithography, laser cutting, and photolithography) allow for surfaces with microstructures like microfluidic channels, sensor arrays, and microneedles to enhance the functionality and sensing accuracy [80].

Based on the above crosslinking mechanisms, functionalized approaches, and manufacturing strategies, hydrogels excel in wound care by integrating biosafety with tunable mechanical flexibility, dynamic adaptability, and functionality. Due to their inherent biocompatibility and biodegradability, hydrogels represent a promising candidate for diverse local and systemic therapeutic applications, owing to their low immunogenicity and tunable degradation kinetics [81,82]. Their mechanical flexibility and robust adhesion enable conformal skin contact for precise signal acquisition in real-time monitoring [83,84]. Beyond their static properties, their dynamic features such as a self-healing ability allow hydrogels to withstand mechanical stress, keeping their functionality during movement [85,86]. In addition, the functionality of hydrogels is enhanced by stimuli responsiveness (e.g., pH, temperature, or light sensitivity), allowing them to dynamically regulate drug release in response to wound conditions [87,88]. In conclusion, these properties make hydrogels multifunctional wound-care platforms capable of bridging passive protection with active wound management.

## 3. Hydrogel-Based Wearable Wound-Monitoring Devices

Hydrogels are ideal materials for wound dressing due to their high water content, biocompatibility, and soft tissue-like structure. By integrating sensing components or stimuli-responsive materials, hydrogel-based wound dressings can monitor the wound’s microenvironment in real time and provide on-demand treatment. In this section, we will introduce various wound biomarkers to evaluate wound situations, including (1) the significance of these biomarkers for wound healing, (2) the technical principles of hydrogel-based sensors for sensing, and (3) an overview of the existing hydrogel-based wound-monitoring devices and their advantages and limitations.

### 3.1. Real-Time Physiological Sensing

#### 3.1.1. Temperature

Temperature is a critical parameter in wearable healthcare devices, enabling real-time physiological monitoring, the early diagnosis of infections, and responsive therapeutic interventions [89,90]. The working principles of hydrogel-based wearable devices are mainly volume phase transitions and electrical responses. For example, thermo-responsive hydrogels (e.g., PNIPAm) can respond to temperature variances to realize volume phase transitions [91], while conductive hydrogels can also respond to temperature, driven by ion migration and conductivity changes [92].

A key challenge in wearable temperature sensing is maintaining its accuracy under mechanical stress, such as bending, stretching, and compression. Wu et al. addressed this problem by developing a deformation-insensitive MXene/clay/PNIPAm (MCP) hydrogel sensor inspired by biological thermoreceptors [93]. The phase transition of the MCP hydrogel is at a threshold temperature (~32 °C), enabling a spike response independent of mechanical strain. Therefore, the MCP hydrogel enables temperature sensing under various deformation conditions (i.e., pressing and 15% stretching), achieving precise temperature monitoring on dynamic surfaces like human skin. Similarly, taking advantage of the thermal responsiveness of PNIPAm hydrogels, Li et al. designed a multi-bioinspired conductive hydrogel patch composed of tannin-grafted gelatin, Ag–tannin nanoparticles, PAAm, and PNIPAm with controlled adhesion, antibacterial, and drug release features, and monitoring functions for intelligent wound management [94]. Interestingly, the photothermal responsiveness of the tannin-grafted gelatin and Ag–tannin nanoparticles allowed for controlled drug release, demonstrating how hydrogel-based wound-care systems can synergize sensing and therapeutic functions.

#### 3.1.2. Oxygen

Oxygen is crucial for cellular metabolism, angiogenesis, and infection prevention during wound healing, making the transcutaneous oxygen pressure (TcpO_2_) a key parameter in assessing wound microcirculation and macrovascular conditions [95]. Hydrogel-based wearable technology provides promising solutions for real-time oxygen sensing due to its excellent porosity and gas permeability, enabling efficient oxygen diffusion and transport [96,97].

For example, Xia et al. introduced a thread-based electrochemical oxygen sensor utilizing silver-coated threads and hydrogel dielectric coatings [98]. The device leveraged the mechanical flexibility of textiles to enable minimally invasive tissue oxygenation monitoring, achieving a high sensitivity detection within the physiological range. In addition, the device showed a robust performance under repeated bending, with a low signal variation (less than 10%). By using the hydrogel-based tissue model, the sensor demonstrated a high spatial resolution, making it suitable for detecting hypoxic regions in deep wound beds. Furthermore, a self-healing, self-adhesive organohydrogel sensor using a PAAm–chitosan double-network structure was developed to further optimize the performance of the oxygen sensor [99]. This sensor exhibited a full detection range (0–100%), an ultralow limit of detection (5.7 ppm), a high sensitivity (0.2%/ppm), and stable operation across extreme temperatures (−18 to 40 °C) and humidity levels (11.3–90.5% RH). These excellent properties are thanks to the incorporation of 1,2-propanediol via solvent replacement to enhance the moisture retention and freezing resistance. In addition, the redox reactions at the hydrogel–electrode interface ensured a high sensitivity and selectivity against interfering gases. Specifically, when the sensor is exposed to a specific concentration of oxygen, the oxygen gains electrons at the cathode, leading to the generation of OH^−^ through a reduction reaction.

#### 3.1.3. Moisture

Moisture monitoring is vital for wound healing because maintaining an optimal moisture level directly influences tissue regeneration and the infection risk [100]. Hydrogel-based wearable devices represent a promising approach for real-time monitoring by exploiting the humidity-responsive capacity of hydrogels, leading to mechanical and electrical characteristics changes [101].

For instance, ion-conductive double-network hydrogel films adjust their ionic conductivity in response to humidity changes [102]. The film is composed of PAAm/carrageenan integrated with PDMS substrate, and it shows a good environmental stability and an ultrahigh sensitivity to humidity, benefitting from the ultrahigh surface-area-to-volume ratio, abundant active sites, and short diffusion distance of the materials. In addition, optical hydrogel sensors further refine their sensitivity through spectral reflection and laser interference, thereby ensuring a rapid responsiveness over a broad humidity range [103]. Specifically, the thickness of the hydrogel layer increases when exposed to air, after which it can be measured with a laser and a broadband light source.

### 3.2. Biochemical Biomarker Analysis

#### 3.2.1. pH

The pH serves as a key indicator of wound infections, with an elevated pH (7.5–9.0) often correlating with bacterial colonization [104]. Optical and electrochemical sensing approaches both enable the real-time, non-invasive detection of the pH, providing critical insights into the infection status, healing progression, and metabolic dysregulation.

**Figure 2:**
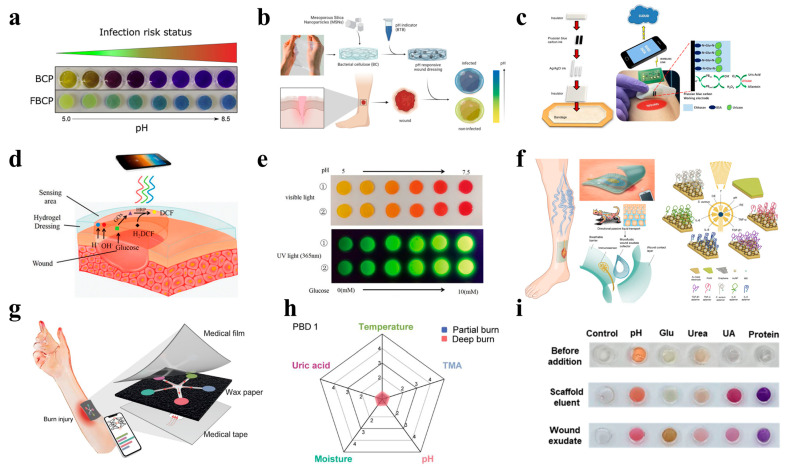
**Wearable hydrogel-based wound-monitoring devices**. (**a**) A pH-monitoring system for an infection risk assessment [105]. Reproduced/adapted with permission from [105], ELSEVIER, 2019. (**b**) The preparation of pH-responsive nanocomposite dressings integrated with a pH-sensitive dye [106]. Reproduced/adapted from [106], ELSEVIER, 2023. (**c**) A wearable smart bandage for uric acid monitoring via wireless communication with computers or smartphones [119]. Reproduced/adapted with permission from [119], ELSEVIER, 2015. (**d**) A PCB hydrogel dressing that enables the dual detection of pH and glucose levels [14]. (**e**) The simultaneous detection results for pH (visible light) and glucose concentrations (UV light) in ① PBS and ② AWE solutions [14]. Reproduced/adapted from [14], WILEY, 2020. (**f**) A schematic illustration and prototype of a multiplexed immunosensing system for chronic wound monitoring [125]. Reproduced/adapted from [125], WELIEY, 2021. (**g**) A schematic of a battery-free colorimetric multiplexed sensor for wound monitoring [127]. (**h**) A real sensor patch resembling a five-petaled flower, with sensing materials and principles for each colorimetric sensor [127]. Reproduced/adapted with permission from [127], ACS, 2023. (**i**) The detection of wound markers in rat wound fluids using a perturbed wound model [126]. Reproduced/adapted from [126], ELSEVIER, 2023.

Optical sensing strategies often leverage colorimetric, fluorescent, or luminescent responses to biochemical changes in the wound microenvironment. For example, Gamerith et al. developed a silane-based coupling method to anchor bromocresol purple onto solid substrates, creating a pH-responsive material that transitions from yellow to green to blue as the pH increases [105]. This system provides high visual contrast to the reddish color of most wound fluids, facilitating rapid infection detection. Clinical validation confirmed a strong association between the pH and the microbial burden, underscoring its diagnostic utility (Figure 2a). To further improve the pH detection resolution, Eskilson et al. engineered bacterial nanocellulose (BC)-based dressings functionalized with mesoporous silica nanoparticles (MSNs) encapsulating a pH-sensitive dye [106]. The BC-MSN nanocomposite achieved a spatiotemporal pH resolution while maintaining an optimal conformability, mechanical strength, and water vapor transmission rates, essential for chronic wound management (Figure 2b).

**Figure 3:**
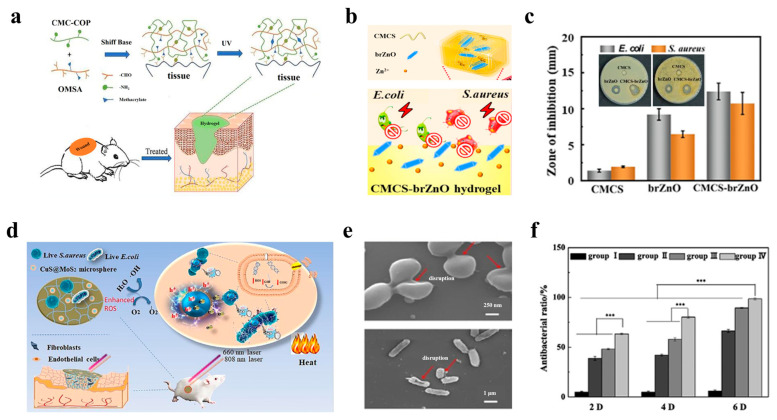
**Wearable hydrogel-based wound dressings with antimicrobial and anti-inflammatory functions.** (**a**) Schematic diagram of the preparation process for collagen peptide-functionalized carboxymethyl chitosan and oxidized sodium alginate bivalent-network hydrogel dressings [132]. Reproduced/adapted with permission from [132], ELSEVIER, 2021. (**b**) Injectable CMCS-brZnO hydrogel dressing with antibacterial activity [133]. (**c**) Comparison of inhibition zones for pristine CMCS, brZnO, and CMCS-brZnO hydrogels [133]. Reproduced/adapted with permission from [133], ELSEVIER, 2022. (**d**) Schematic illustration of a bifunctional hydrogel incorporated with CuS@MoS microspheres for disinfection and enhanced wound healing [134]. (**e**) SEM morphology of bacterial biofilms [134]. (**f**) Antibacterial rates after 2, 4, and 6 days of treatment [134]. *** *p* < 0.001.Reproduced/adapted with permission from [134], ELSEVIER, 2020.

Electrochemical sensing offers the real-time quantification of biomarkers compared to optical sensing. For instance, by integrating laser-scribed indium tin oxide (ITO) electrodes with wound dressings, Rahimi et al. developed a flexible, transparent pH sensor with polyaniline-functionalized working electrodes (Figure 3e). The sensor achieved a sensitivity of −55 mV/pH (pH of 4–10), enabling real-time wound inspection [107]. In addition, a biocompatible NFC tag facilitated a wireless smartphone readout, realizing real-time pH detection for bacterial infections.

#### 3.2.2. Glucose

Glucose metabolism plays an important role in wound healing, in which glucose transporters (GLUTs) and related metabolic pathways (e.g., glycolysis and lactate production) provide energy for cells to support angiogenesis and extracellular matrix remodeling [108]. However, an abnormal glucose metabolism (e.g., hyperglycemia) can lead to oxidative stress and the release of inflammatory factors, further interfering with wound healing [109,110]. There are two main types of methods for detecting the glucose concentration: enzymatic and non-enzymatic methods. Non-enzymatic methods employ fluorescent molecular imprinting technology combined with fluorescein Tt, achieving the quantitative detection of the glucose concentration through changes in fluorescence signals, which is highly selective [111]. In addition, glucose-responsive hydrogels are used to monitor glucose levels in wound exudate by enzymatic approaches, providing evaluable support for diabetic wound management [112]. For example, a hydrogel microneedle array-based transdermal dressing system composed of a photocrosslinkable hydrogel of gelatin methacrylate (GelMA) with a pH-responsive nanogel and glucose oxidase (GOx) was designed for monitoring the glucose levels in chronic wounds [113]. The composite hydrogel showed a fast response and a high sensitivity to glucose levels in the physiological range, mainly due to the ionization of the pH-responsive component and the proton balance.

#### 3.2.3. Uric Acid

Uric acid is the end product of purine metabolism, and its concentration changes significantly during wound healing [114]. Studies have shown that uric acid levels increase due to cell death and inflammatory responses during wound healing, and are associated with the increased expression of inflammatory factors (e.g., IL-1β and IL-6) [115]. In addition, in patients with chronic venous ulcers, the uric acid levels are significantly higher than in healthy skin, indicating that uric acid can be used as an indicator of wound severity [114].

In recent years, a variety of uric acid-based sensors have been developed for the real-time monitoring of wound healing [116,117]. For example, an enzymatic uric acid biosensor can continuously provide valuable information about wound healing by detecting the uric acid levels. By integrating urate oxidase with a PVA hydrogel, the biosensor can detect uric acid in 0.5–50 μL of wound fluid, realizing highly sensitive detection [118]. Furthermore, Kassal et al. designed a screen-printed amperometric biosensor on a wound dressing, immobilizing uricase and Prussian blue for uric acid detection at low working potentials (−0.1 V vs. Ag/AgCl) [119]. The biosensor exhibited an excellent linearity (R^2^ =0.9985) over the full physiological concentration range and interfaced with a wearable potentiostat for wireless data transmission via RFID/near-field communication (NFC). Clinical validation showed a robust performance under mechanical stress (80 bends), addressing the challenge of sensor durability in mobile patients (Figure 2c).

#### 3.2.4. Cytokines

Cytokines are a promising biomarker of systemic inflammatory responses, because they regulate all stages of wound healing, while a dysfunction in cytokine production can cause non-healing inflammation [120]. IL-1, IL-2, IL-6, IL-8, and TNF-α are important proinflammatory cytokines that accelerate wound healing by activating related signaling pathways and promoting fibroblast migration, angiogenesis, and collagen deposition [121,122]. Variation in the concentrations of IL-6 and TNF-α can reflect the level of inflammation and the healing stage of the wound. For example, during acute wound healing, the IL-6 and TNF-α levels increase significantly, and they gradually decrease in the later stages of healing [123]. By utilizing hydrogel sensors integrated with aptamers, these inflammatory factors can be detected effectively by fluorescent signals. For example, a flexible, wearable biosensor based on a graphene field-effect transistor was designed, which incorporates aptamer-functionalized graphene as the conductive channel. The sensor enables the highly sensitive monitoring of cytokine levels (TNF-α and IFN-γ) with limits of detection down to 2.75 and 2.89 pM in human bodily fluids [124].

### 3.3. Multiplexed Biomarker Analysis

A multiplexed biomarker analysis can provide a comprehensive, real-time snapshot of a wound microenvironment, especially for chronic wounds (e.g., diabetic foot ulcers, venous leg ulcers, pressure injuries). These wounds are complex and affected by multiple interacting factors (e.g., infections, inflammation, and metabolic dysregulation), while single biomarkers often provide incomplete information.

Diabetic ulcers require the simultaneous monitoring of the pH and glucose to address hyperglycemia-driven complications. For example, Zhu et al. designed a zwitterionic poly-carboxybetaine (PCB) hydrogel co-encapsulating phenol red (a pH indicator), glucose oxidase (GOx), and horseradish peroxidase (HRP) [14]. The anti-biofouling PCB matrix enhanced enzyme stability, increasing HRP’s catalytic efficiency by 5.5-fold compared to free enzymes (Figure 2d). A smartphone-based RGB analysis enabled the quantitative tracking of the pH (4–8) and glucose (0.1–10 mM), while the moist environment of the hydrogel accelerated healing in diabetic wounds (Figure 2e).

The heterogeneous nature of chronic wounds demands the multiplexed detection of inflammatory cytokines, pathogens, and physicochemical parameters. By integrating immunosensors with microfluidic technology, Gao et al. created a flexible immunosensor array for the simultaneous profiling of TNF-α, IL-6, IL-8, TGF-β1, *S. aureus*, the pH, and the temperature (Figure 2f). This platform combines microfluidic exudate collection with graphene-based immunosensors and a smartphone interface, achieving low detection limits for cytokines (e.g., 30 ng/mL for IL-6) and a good monotonicity for *S. aureus* (R^2^ = 0.9712). In trials on patients with venous leg ulcers, the system correlated TNF-α, IL-6, IL-8, TGF-β1, *S. aureus*, the pH, and the temperature with delayed healing over 5 weeks, underscoring its diagnostic potential [125]. In addition, by taking advantage of optical sensing strategies, a carbon dot-doped agarose hydrogel sensor array embedded in polydimethylsiloxane (PDMS) for the simultaneous colorimetric detection of the pH, glucose, urea, uric acid, and the total protein was engineered [126] (Figure 2g). To enhance the stability and signal fidelity of the enzymatic sensor, biogenic carbon dots were introduced, synthesized from amino acid–polymer precursors. Clinically validated in rat wound models, the array distinguished healing and non-healing wounds via distinct color patterns, achieving a high accuracy in the detection of all biomarkers (Figure 2h).

Applying artificial intelligence algorithms to the complex data analysis can provide accurate prognostic predictions and optimized treatment guidance, revolutionizing traditional chronic wound management. For example, Zheng et al. developed a battery-free, AI-enabled multiplexed sensor patch (PETAL) for in situ wound assessments [127]. The PETAL sensor incorporates five wax-printed colorimetric sensors on a paper panel, targeting the temperature, the pH, trimethylamine (TMA), uric acid, and the moisture (Figure 2i). A convolutional neural network (CNN) was used to analyze smartphone-captured sensor images, achieving a 97% accuracy in classifying the healing versus non-healing status in rat burn and perturbed wound models. This system enables the early detection of complications and triggers timely clinical interventions, representing a leap toward autonomous, point-of-care wound diagnostics.

## 4. Hydrogel-Based Wound Healing and Therapeutic Platforms

Based on the moist healing theory, hydrogel-based wound dressings can significantly accelerate wound healing. There are mainly three aspects to the advantages of hydrogel-based wound dressings compared to traditional ones. Firstly, they can maintain the humidity of the wound microenvironment due to their 3D hydrophilic network, promoting cell migration and angiogenesis [128]. Secondly, their structure mimics the ECM, providing oxygen and water exchange and absorbing excess exudate to prevent secondary damage caused by the adhesion of traditional dressings [129]. Finally, the excellent degradability and biocompatibility of hydrogels make them an ideal therapeutic platform loaded with antibacterial, anti-inflammatory, or antioxidant components to achieve rapid wound healing [130].

### 4.1. Antimicrobial and Anti-Inflammatory Functions

The antimicrobial and anti-inflammatory functions of wound dressings are critically important for wound healing because they directly address two of the most significant barriers for efficient tissue repair (i.e., infection and dysregulated inflammation). Hydrogel-based wound dressings employ diverse antimicrobial strategies that provide a physical barrier and the controlled release of bioactive agents to fight infections and dysregulated inflammation [131]. For instance, He et al. developed a double-network hydrogel composed of collagen peptide-functionalized carboxymethyl chitosan (CS) and oxidized methacrylate sodium alginate (SA), enhancing both the mechanical robustness and the therapeutic efficacy [132]. Specially, aldehydes on the oxidized methacrylate SA can quickly crosslink with amino groups on the CS via a Schiff base reaction, and they can then form a CS/SA hydrogel by photo-crosslinking. This system can accelerate wound healing in vivo by regulating inflammatory cytokine levels, promoting collagen deposition, and enhancing vascularization in full-thickness skin defect models (Figure 3a).

The antimicrobial efficacy can be further augmented through the incorporation of inorganic nanostructures. For example, by embedding fusiform-like zinc oxide nanorods (brZnO) into carboxymethyl chitosan (CMCS), Hu et al. engineered an injectable carboxymethyl chitosan–fusiform zinc oxide nanorod (CMCS-brZnO) hydrogel [133] (Figure 3b), which presented an excellent antibacterial performance by releasing Zn^2+^ ions at minimal inhibitory concentrations (0.0125 mg/mL against *E. coli* and 0.025 mg/mL against *S. aureus*) (Figure 3c). In addition, the hydrogel wound dressing exhibited rapid self-healing, tissue adhesion, and injectability even in irregular wounds, demonstrating synergy between organic and inorganic microstructures in promoting wound healing.

Innovative photoresponsive hydrogels have also emerged to address multidrug-resistant infections. For instance, a CuS@MoS_2_ microsphere-incorporated PVA hydrogel was synthesized, rendering excellent antibacterial properties under photothermal (808 nm near-infrared) and photodynamic (660 nm visible light) therapies (Figure 3d). Under co-irradiation, the hydrogel generated hyperthermia and ROS, achieving an antibacterial efficiency of 99.3% for Escherichia coli (*E. coli*) and 99.5% for Staphylococcus aureus (*S. aureus*) within 15 min (Figure 3e,f). Beyond rapid pathogen killing, this system also promoted vascularization by upregulating hypoxia-inducible factor 1α (HIF-1α) and VEGF, thereby mitigating chronic inflammation and accelerating wound healing [134].

### 4.2. Stimuli-Responsive Drug Delivery

Stimuli-responsive hydrogels have emerged as advanced platforms for controlled drug delivery in wound treatment, enabling a dynamic adaptation to the pathophysiological microenvironment of chronic wounds [135,136]. These platforms mainly respond to endogenous or exogenous triggers (such as the pH, temperature, glucose levels, light, or magnetic fields) to achieve the on-demand release of therapeutic agents, thereby enhancing the wound-healing efficacy.

**Figure 4:**
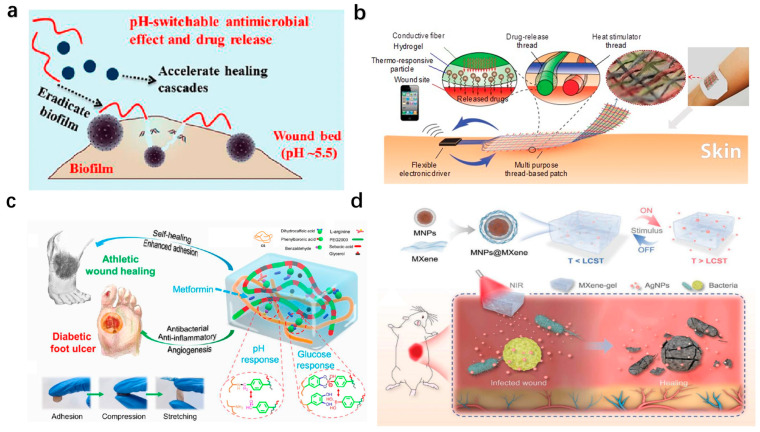
**Wearable hydrogel-based wound dressings with stimuli-responsive drug-delivery functions.** (**a**) pH-switchable antimicrobial nanofiber networks eradicating biofilms and rescuing stalled healing in chronic wounds [137]. Reproduced/adapted with permission from [137], ACS, 2019. (**b**) Multipurpose thread-based patch for transdermal drug delivery, featuring a hydrogel layer with thermo-responsive particles coated on a flexible thread-based heater [17]. Reproduced/adapted with permission from [17], WILEY, 2017. (**c**) pH/glucose dual-responsive metformin-release hydrogel dressings with adhesion and self-healing via dual-dynamic bonding for athletic diabetic foot wound healing [138]. Reproduced/adapted with permission from [138], ACS, 2022. (**d**) NIR-responsive AgNP-loaded MXene-based hydrogel system [139]. Reproduced/adapted with permission from [139], WILEY, 2022.

Based on the pH-responsive mechanism, Wang et al. developed a pH-switchable antimicrobial hydrogel composed of self-assembled octapeptide nanofiber networks, which destabilize under the acidic pH levels characteristic of infected chronic wounds (Figure 4a). In addition, this pH-dependent release mechanism activates antimicrobial peptide-like activity, disrupting bacterial membranes and eradicating biofilms. Furthermore, this hydrogel synergistically releases a photothermal agent (i.e., cypate) and a procollagen component (i.e., proline), achieving combined biofilm elimination and collagen deposition. In in vivo experimental models, this network enabled the complete wound healing of MRSA-biofilm-infected wounds within 20 days, showing great potential as promising chronic wound dressings [137].

Thermal responsiveness has also been widely exploited to achieve controlled drug delivery. For example, Mostafalu et al. engineered a textile-based dressing integrating thermo-responsive hydrogel fibers with embedded electrical heaters (Figure 4b). Each fiber, loaded with distinct therapeutics (e.g., antibiotics and VEGF), enables on-demand release via localized heating, demonstrating precise temporal control over antibiotic and VEGF release. The efficiency of the dressing was tested in diabetic mouse models, showing great potential to induce angiogenesis and then promote wound healing [17].

Dual-responsive systems targeting complex wounds, such as diabetic foot ulcers, are critical. By employing a pH/glucose dual-responsive mechanism, Liang et al. designed a pH/glucose dual-responsive hydrogel via Schiff base and phenylboronate ester bonds (Figure 4c). The hydrogel exhibited self-healing, adhesion, and the sustained release of metformin and graphene oxide (GO) in response to elevated glucose and an acidic pH. In type II diabetic rat models, the hydrogel reduced oxidative stress, suppressed inflammation, and enhanced angiogenesis [138]. To address deep chronic wounds, Yang et al. developed a multi-stimuli-responsive MXene-based hydrogel combining photothermal (808 nm NIR) and magnetic responsiveness (Figure 4d). The hydrogel integrated MXene-wrapped magnetic colloids within a PNIPAm–alginate dual network, enabling controlled drug release under external light or magnetic fields. In rat models of full-thickness cutaneous and subcutaneous infected wounds, targeted drug delivery to deep tissues was realized, minimizing off-target toxicity while promoting vascularization and infection control [139].

### 4.3. Mechanical and Structural Support

Advances in biomechanics and mechanobiology have revealed that mechanical factors play a pivotal role in wound healing. Mechanically robust and structurally adaptive hydrogels have garnered significant attention as advanced wound dressings, addressing the limitations of conventional passive systems by integrating dynamic mechanical support with bioactive functions [140,141].

**Figure 5:**
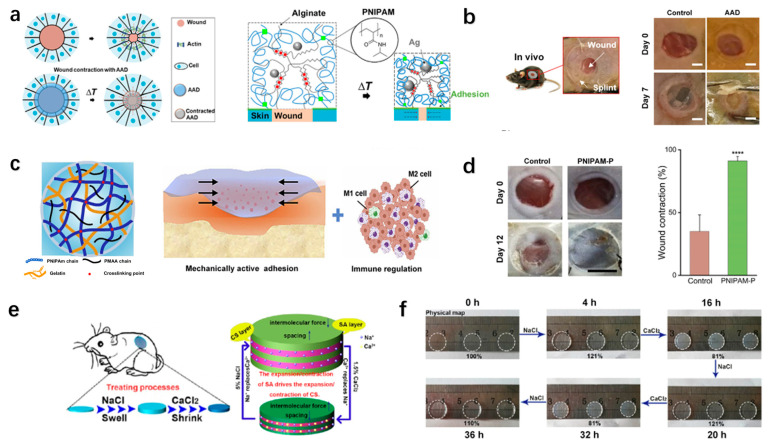
**Wearable hydrogel-based wound dressings with mechanical and structural functions.** (**a**) Bioinspired design of active adhesive dressing (AAD) for wound contraction, composed of PNIPAm, alginate, and AgNPs [142]. (**b**) In vivo wound-healing outcomes with AAD application [142]. Reproduced/adapted from [142], Science, 2019. (**c**) Mechanically active adhesive and immune-regulatory dressings (PNIPAm-P hydrogel) for accelerated wound closure [143]. (**d**) Wound repair efficacy of PNIPAm-P hydrogel in a mouse model [143]. **** *p* < 0.0001. Reproduced/adapted with permission from [143], Cell Press, 2021. (**e**) Ion-excited mechanically active self-assembling membranes inhibiting microbial infection and promoting rapid healing via expansion–contraction mechanisms [144]. (**f**) Hydrogel expansion and contraction images at sequential time points [144]. Reproduced/adapted with permission from [144], ACS, 2021.

Inspired by embryonic wound contraction, a mechanically active adhesive dressing using thermo-responsive tough adhesive hydrogels was developed, exhibiting a high stretchability (>1400% strain), tissue adhesion (fracture energy, ~500 J/m^2^), and antimicrobial properties (Figure 5a). Upon exposure to skin temperatures, the hydrogel undergoes controlled contraction, reducing the wound area by up to 45% in rodent models (Figure 5b). In addition, finite element modeling validated the stress distribution and contraction dynamics, confirming their efficacy in accelerating wound healing [142]. The active promoted healing strategy eliminates the need for exogenous drugs, providing a potent mechanobiological approach for wound closure.

Expanding on active mechanical modulation, Hu et al. developed a temperature-sensitive hydrogel with a semi-interpenetrating network (semi-IPN) of poly(methacrylic acid) (PMAA), which adhered strongly to wounds and contracted actively upon hydration, mimicking physiological wound closure (Figure 5c). Notably, the PMAA component synergistically enhanced angiogenesis and modulated inflammatory responses by downregulating TNF-α and IL-6 while upregulating IL-10 [143]. In porcine wound models, the hydrogel reached a healing rate close to 60% compared to passive dressings, emphasizing the dual role of mechanical activity and immune regulation (Figure 5d).

In addition to thermo-responsive mechanical contraction, an ion-responsive mechanism can be employed to develop active promoted wound dressings. For instance, Dong et al. engineered ion-responsive self-assembling membranes (LBLSMs) via Na^+^/Ca^2+^-mediated layer-by-layer assembly (Figure 5e). The LBLSMs exhibited tunable mechanical and optical properties under ionic stimulation (Figure 5f). This mechano-active behavior promoted fibroblast proliferation and collagen deposition in rodent models, achieving 100% wound closure within 12 days. Additionally, the membrane demonstrated an inherent antibacterial efficacy, attributed to cation-mediated membrane disruption, without requiring exogenous antimicrobial agents [144].

## 5. Hydrogel-Based Integrated Systems: Combining Monitoring and Therapy

The integration of real-time diagnostic capabilities with on-demand therapeutic interventions represents a paradigm shift in wound care, addressing the dynamic and complex nature of chronic or infected wounds. However, the real challenge is how to make them work together seamlessly, because the feedback loop framework functions when making two modules integrated rather than just bundled. To build a closed-loop framework with real-time diagnostics and guided therapeutics, there are three core components that should be considered: biosensors, analytical engines, and therapeutic modules. Then, the closed loop can be formed by applying the optimal treatment Based on real-time sensor-derived wound parameters quantified through computational analysis.

**Figure 6:**
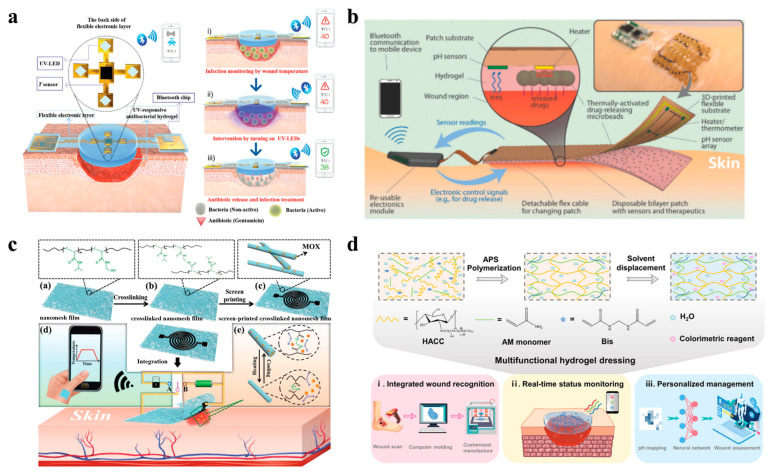
**Wearable hydrogel-based wound dressings combining monitoring and therapy functions.** (**a**) A smart flexible electronics-integrated wound dressing, consisting of a polydimethylsiloxane-encapsulated flexible electronic layer and a UV-responsive antibacterial hydrogel [147]. Reproduced/adapted from [147], WILEY, 2020. (**b**) An automated smart bandage comprising an array of flexible pH sensors and a flexible heater to trigger thermo-responsive drug carriers containing antibiotics [148]. Reproduced/adapted with permission from [148], WILEY, 2018. (**c**) A flexible and breathable on-skin electronic device featuring temperature-sensing capabilities and temperature-sensitive, on-demand drug release [149]. Reproduced/adapted with permission from [149], WILEY, 2021. (**d**) A multifunctional hydrogel as a wound dressing for intelligent wound monitoring, including wound recognition, real-time status monitoring, and personalized wound management [150]. Reproduced/adapted with permission from [150], ELSEVIER, 2022.

Advanced hydrogel-based systems now combine biosensing, data processing, and stimuli-responsive drug delivery to enable closed-loop, personalized wound management, bridging the gap between passive dressings and intelligent therapeutic platforms [145,146]. For example, Pang et al. developed a smart flexible electronics-integrated dressing that featured a bilayer architecture, including an upper layer of PDMS-encapsulated flexible electronics with a temperature sensor and UV LEDs and a lower UV-responsive antibacterial hydrogel layer, enabling real-time temperature monitoring for early infection detection and on-demand antibiotic release via in situ UV irradiation (Figure 6a). In vitro studies demonstrated its ability to detect pathogenic colonization and trigger localized treatment, achieving an obvious reduction in the bacterial load while maintaining a high flexibility and biocompatibility [147]. This integration design exemplifies the potential of combining biosensing with spatiotemporal control over antimicrobial therapy. Expanding on the closed-loop feedback system, Mostafalu et al. engineered a multifunctional smart bandage integrating pH and temperature sensors, a thermo-responsive hydrogel loaded with drug carriers, and a microcontroller for data-driven treatment (Figure 6b). The hydrogel releases therapeutics (e.g., antibiotics, growth factors) via electronically controlled heating, guided by sensor feedback on the wound status. The results of CFU experiments indicated that this platform can accelerate wound healing by synchronizing anti-infection and pro-angiogenic therapies according to the dynamic wound microenvironment [148].

By focusing on improving the wearability and wireless data transmission of wearable hydrogel wound dressing systems, Gong et al. designed a breathable nanomesh electronic device using a crosslinked electrospun thermo-responsive polymer loaded with moxifloxacin hydrochloride (MOX) (Figure 6c). The conductive nanomesh functioned as a temperature sensor and a flexible heater to trigger antibiotic release upon infection. In addition, its high porosity ensured oxygen and moisture permeability, which iscritical for minimizing maceration [149]. Furthermore, the analytical engine can be assisted by an AI algorithm, realizing intelligent wound management. For instance, a multifunctional hydrogel dressing incorporating pH-responsive colorimetric sensing and a CNN-based machine learning algorithm was created (Figure 6d). The CNN algorithm, trained on wound image datasets, achieved a 94.47% accuracy in predicting the healing progress, enabling automated, personalized treatment recommendations [150]. Therefore, such a system merges biomaterial design with an AI-driven data analysis, providing a potential benchmark for next-generation smart wound care.

In conclusion, these integrated systems highlight the combination of material science, bioelectronics, and data analyses in modern wound management. By integrating real-time biosensors, adaptive drug delivery, and AI-powered data analyses and decision-making, hydrogel-based wound dressings are evolving into intelligent wound-care platforms. In the future, advancements are needed in multi-parameter sensing, high-efficiency therapeutic operations, and wireless data transmission to further enhance the clinical outcomes for chronic wounds.

## 6. Challenges and Future Perspectives

### 6.1. Challenges

Despite significant advancements, hydrogel-based wound dressings face two challenges that limit their abroad application, mainly in fabrication technology and clinical applications. For fabrication technology, the signal accuracy, long-term stability, and power supply of devices should be considered to adapt the dynamic wound microenvironment. For clinical applications, biocompatibility is important to ensure the healing outcomes. In addition, due to variation in wound shapes, sizes, and locations, personalized wound treatment should be provided for individual patients to meet their specific wound-care demands.

For fabrication technology, the first challenge lies in ensuring the signal accuracy and reliability in dynamic wound environments, where factors such as a variable pH, temperature fluctuations, and the exudate composition can interfere with embedded sensors [151]. For instance, electrochemical sensors for pH may suffer from false readings due to protein adsorption or ionic interference, leading to inaccurate signals and, thus, affecting the assessment of the wound condition [152,153]. Additionally, realizing stability remains challenging, because long-term mechanical stress during patient movement further results in the structural fatigue of the devices. The performance of the hydrogel (e.g., moisture retention, antibacterial properties) may also degrade over extended periods [154]. Lastly, a power supply and the miniaturization of electronic components should be considered for convenience in practical applications [155].

For clinical applications, adverse effects associated with the materials and structures remain a substantial concern. For instance, many hydrogels exhibit a low cytotoxicity in vitro, but their long-term interaction with immune cells can trigger unintended immune responses, including chronic inflammation, granuloma formation, or excessive fibrosis, leading to undesired healing outcomes. Therefore, biocompatibility is crucial for realizing a long-term diagnosis and therapy. Moreover, the heterogeneity of chronic wound microenvironments, including variations in biofilm composition, oxidative stress, and hypoxia, complicates the design of a treatment strategy [156,157]. For example, a hydrogel optimized for diabetic foot ulcers may fail in venous leg ulcers due to differences in the protease activity or microbial colonization [158]. Lastly, translating preclinical success to human trials remains challenging. The efficacy of most hydrogel wound dressings is demonstrated in rodent models and not in human patients, and obvious differences exist in wound physiology between them.

### 6.2. Future Perspectives

Firstly, to realize dual monitoring and therapy, building hybrid systems is necessary, which involves integrating inorganic nanoparticles (e.g., MXenes, quantum dots) with biopolymers, thus enhancing the mechanical and optical properties [159]. In addition, developing multifunctional hydrogels with various properties (e.g., stimuli-responsive, self-healing, and conductivity properties) is important in the future. For example, enzyme-mimetic hydrogels with conductivity enable ROS scavenging and real-time electrophysiological monitoring [160], in which ROS levels can be detected by enzyme-mimetic hydrogels, and impedance changes can be acquired from conductive polymers.

Secondly, assisted by AI technology, data can be analyzed efficiently to revolutionize wound care, which enhances the current wound-care systems significantly [161,162]. Specifically, machine learning algorithms can analyze complex, real-time data streams to dynamically predict wound conditions. In addition, sensor data autonomously trigger the optimal treatment (e.g., drug release, electrical stimulation etc.), building a closed-loop feedback system and representing the next frontier in personalized wound management [163]. For instance, AI-driven wound dressings could adapt treatment protocols in real time based on patient-specific healing patterns, thus optimizing the wound-healing outcomes [150].

Lastly, future perspectives emphasize intelligent and green wound-care systems that merge biocompatible materials, advanced fabrication technology, and wireless data transmission. Emerging solutions, such as bioenergy-harvesting devices (e.g., triboelectric nanogenerators) or wireless charging, are being explored to enable self-sustaining operation [164]. In addition, advances in 3D bioprinting while employing biocompatible materials may enable the construction of ecosystems for patient-specific wound healing [165].

## 7. Conclusions

Hydrogel-based wearable technologies have emerged as innovative solutions in modern wound care, representing a paradigm shift from passive to active wound management. The unique properties of hydrogels make them particularly suitable for addressing complex wound healing. This review introduced the fundamentals of hydrogels, from their basic biomaterials to functional platforms integrating real-time monitoring, targeted therapy, and integrated diagnoses with treatments. Specially, the development of wearable sensors incorporated into hydrogel matrices has significantly advanced wound assessment capabilities through the continuous monitoring of both the physiological parameters and the biochemical markers, providing valuable guidance for the early detection of complications. Therapeutic hydrogel applications have expanded well beyond basic antimicrobial functions to include drug delivery and mechanically promoted contractions that actively facilitate tissue repair. The integration of these capabilities into hydrogel-based wound-care systems demonstrates the exciting potential for a closed-loop, personalized wound management strategy, while challenges in engineering and clinical application persist. By discussing the current limitations and future directions, next-generation hydrogel-based wound dressings will transition from passive bandages to proactive, intelligent systems.

## Data Availability

Not applicable.
